# How Do Children “Think outside the Box”? Fluid Intelligence and Divergent Thinking: A Moderated Mediation Study of Field Dependent-Independent Cognitive Style and Gender

**DOI:** 10.3390/children11010089

**Published:** 2024-01-11

**Authors:** Marco Giancola, Massimiliano Palmiero, Maria Chiara Pino, Marta Sannino, Simonetta D’Amico

**Affiliations:** 1 Department of Biotechnological and Applied Clinical Sciences, University of L’Aquila, 67100 L’Aquila, Italy; marco.giancola@univaq.it (M.G.); mariachiara.pino@univaq.it (M.C.P.); marta.sannino@graduate.univaq.it (M.S.); 2 Department of Communication Sciences, University of Teramo, 64100 Teramo, Italy; mpalmiero@unite.it

**Keywords:** fluid intelligence, creativity, field dependent-independent cognitive style, childhood, moderated mediation

## Abstract

The interplay between fluid intelligence (Gf) and divergent thinking (DT) has widely characterized current research in the psychology of creativity. Nevertheless, the evidence on the main factors involved in this association during childhood remains a matter of debate. Present research has addressed the interplay between Gf and DT, exploring the mediating role of a field dependent-independent cognitive style (FDI) and the moderating effect of gender in 101 children (M_age_ = 8.02; SD_age_ = 1.43). Participants carried out Raven’s Colored Progressive Matrices, the Children Embedded Figure Test, and the Alternative Uses Task. The results revealed the mediating effect of FDI in the association between Gf and DT, providing evidence that this cognitive style represents a function of controlled mental processes underpinned by Gf, which are useful to thinking divergently. In addition, the findings reported that the interplay between FDI and DT was moderated by gender, suggesting that the impact of FDI on DT was stronger among boys. Through a multidimensional approach, these current research findings provide further insight into the primary children’s factors involved in the ability to find alternative solutions and think divergently.

## 1. Introduction

Divergent thinking (DT) reflects the ability to solve ill-defined problems, break down old schemes, and be as original as possible [1,2]. Past research has suggested that DT is the core of creativity, since it is critical in explaining variance in people’s creative achievements and production through the generation of remote and distal concepts [3,4,5]. In addition, the ability to think divergently is customarily conceptualized in terms of (1) fluency, which entails the quantity of ideas generated; (2) flexibility, which comprises the quantity of categorical shifts; (3) originality, which includes the quality of ideas [2]. All indices of DT tend to be highly correlated, and fluency seems to account for substantial variance in originality that, however, was found to have theoretical ties to creativity [6,7]. In addition, research indicated that engaging in divergent activities underpins children’s thoughts and emotions expression, as well as building new forms of knowledge [8]. Despite its critical role during childhood, evidence on the developmental trajectories of DT is elusive. Indeed, even though DT seems to emerge early and gradually grow with “slumps” around ages 5, 9, and 12, the findings widely vary. Some studies revealed an increase of DT across early childhood, whereas others failed to observe developmental changes (see, for a review [9]). Nevertheless, despite potential continuities or discontinuities in DT development, there is wide agreement that DT denotes a complex goal-oriented process supported by the executive system since the early stages of life [10].

Executive functions (EFs) are often conceptualized as a set of cognitive processes which tend to develop rapidly during childhood and then typically continue to grow at a lower rate until early adulthood [11]. According to Diamond’s hierarchical model [12], EFs comprise the core executive functions (CEFs) and the higher executive functions (HOEFs). The CEFs include (1) working memory, which allows for holding and manipulating information in mind; (2) inhibition, which entails the ability to override an automatic response, and (3) cognitive flexibility, which allows for switching between different types of thinking. In turn, the HOEFs cover (1) planning, which depicts the ability to set up plans to reach a goal; and (2) fluid intelligence (Gf). This latter function involves reasoning, problem solving, as well as the ability to understand the relationships among different concepts mainly in novel, demanding, and ambiguous or ill-defined situations [13]. This evidence has led to the discovery of the critical role of Gf during the generation of divergent ideas (see, for a meta-analysis [14]). Different authors argued that Gf is critical when individuals attempt to adapt novel circumstances and solve problems using complex reasoning, generating strategies for inhibiting or shifting thought away from standard responses, maintaining the focus during the idea generation, as well as evaluating the effectiveness of the ideas generated [15,16,17]. Although this notion aligns with a recent meta-analysis [14] in which Gf was found to be modestly correlated with DT (*r* = 0.23), only a handful of research has explored the association between Gf and DT in children. Shi et al. [18], in a sample of children (11–13 years old), found that Gf was positively associated with fluency, flexibility, and originality of visual DT, also indicating that the breakpoint of these relationships occurred at the IQ of 110.10. Similarly, Krumm et al. [19], exploring the impact of EFs in a sample of 8–13 years old children, revealed positive correlations between visual and verbal DT and different facets of intelligence, including Gf, crystalized, and general intelligence. Despite this poor evidence on childhood, mounting research from adolescent and adult samples indicated positive associations between Gf and different indices of DT, substantiating the involvement of goal-oriented cognitive processes in the ability to think divergently [20,21].

Aside from its role in DT, Gf was also found to be pivotal in affecting field dependent-independent cognitive style (FDI). This latter denotes a stable and habitual information processing strategy, which lies in the notion that people usually show different degrees of dependence on the surrounding context [22]. FDI involves two attitudes, that is field dependence and field independence [23]. People with a high degree of field independence prefer to process information analytically, deal well with details, and can separate simple elements from the general context [21]. By contrast, individuals with a high field dependence usually adopt holistic information processing, showing difficulties in perceiving and identifying details within complex contexts [24]. Previous studies demonstrated that FDI is closely associated with EFs, including both CEFs and HOEFs. For instance, some studies indicated that FDI denotes the functioning of the central executive system [25]. Similarly, other studies suggested that the active reasoning components of Gf allows the analytical information processing underpinning field independence [26,27]. Accordingly, the association between Gf and field independence strategies has been also demonstrated across life spans and different cultures [28,29]. For instance, Tinajero–Páramo [29] found similar correlation between children’s Gf and field independence in both boys and girls (*r* = 0.49 for boys and *r* = 0.41 for girls).

Additionally, previous research showed that FDI affects individual ability to think divergently, depicting field independence as a pivotal factor that allows individuals to “think outside the box” [30]. Indeed, whereas individuals with a degree of field dependence are highly conformist, socially sensitive, interpersonally goal-oriented and dependent on cultural pressures, people with high degrees of field independence are usually more flexible, open-minded and able to break down old schemas as well as routine [31,32]. This evidence has been widely confirmed in adolescence and adulthood, whereas the association between FDI and DT in children remains extremely underexplored (see, for review [33]). Indeed, only one study addressed the association between FDI and DT, highlighting that the positive association between field independence and DT occurs only when children had high scholastic attitude [34].

Furthermore, two recent studies indicated that FDI is implicated in the relationship between Gf and DT in adolescents and adults [35,36]. For instance, Giancola and colleagues [36] found that the mediating effect of FDI in the association between young adults’ Gf and individual creative potential as captured in terms of DT, convergent thinking, and creative personality. A related study [35] found an analogous mechanism in adolescence, when FDI is implicated in the interplay between Gf and the only ability to think divergently. Overall, both studies indicated that the high cognitive complexity and the strong disposition toward the solution of problems in an analytical fashion, underpinned by field independence, promote the effect of Gf on DT. Thus, the field independence cognitive style reflects controlled mental processes that are underpinned by Gf and are used to think divergently. The core mechanism shared by Gf, FDI, and DT is related to the ability to process information analytically. In this vein, DT is conceived as being based on conscious goal-directed and sub-goal-directed strategies [10], that allow the generation and the evaluation of new ideas (e.g., the cardboard box can be used as a cover laying it out) with respect to the task constraints imposed by the task goal. This means that DT also involves analytic processes and rationality [37], that is driven by Gf and plausibly by FDI. Overall, this evidence aligns with the investment theory of creativity [38], which posits that interconnected and interdependent analytical resources (i.e., Gf and FDI) are necessary for relevant creative performance. On the one hand, Gf supports the evaluation of the usability and functionality of new ideas [16]. On the other hand, FDI allows for managing Gf during idea generation [35]. According to this notion, DT implies both computational efficiency of Gf as well as the ability to employ cognitive resources underpinned by FDI in order to generate divergent ideas [39].

In addition, this mechanism seems to survive also in adolescence [35], a developmental period characterized by deep neurodevelopmental changes, which affect overall cognition and both Gf and DT [40,41].

Based on the logic behind these two studies, the current research aimed to provide further evidence of the implication of FDI in the link between Gf and DT in a sample of children. Indeed, research on the developmental trends of cognitive styles demonstrated that FDI does not remain static, but changes as a function of age [42,43]. This means that age differences in FDI appear mostly between age groups of 7–8 and 8–9 years (e.g., [44]), and maximally between childhood and adolescence (10–15 years of age) [45]. Interestingly, performance in FDI tasks tends to plateau around the age of 17 years (e.g., [43,46]).

Additionally, the current research study sought to investigate the involvement of gender as a moderator in the interplay between FDI and DT. We specifically focused on this relationship given that gender differences are robust in FDI rather than in Gf and DT [45,46,47,48]. Specifically, previous research indicated that boys generally tend to be more field independent than girls [49,50]. This is not surprising given that autonomy and analytic thinking, two main attributes of field independence, are traditionally masculine, and consequently, they may be more socially reinforced in males [51,52]. In contrast, intuitive or global/holistic thinking, two main attributes of field dependence, are traditionally feminine, and consequently, they may be more strongly reinforced in females. This means that it is possible that boys increase the effect of the independent cognitive style on DT, whereas girls increase the effect of the dependent cognitive style on DT. Such a moderating gender effect does not show up including Gf in the interplay because of the contradictory results on gender differences in Gf. Additionally, given that FDI changes across years, whereas Gf appears more pre-determined, the idea to explore the impact of gender differences in the relationships between FDI and DT during a critical developmental stage is intriguing.

**Figure 1:**
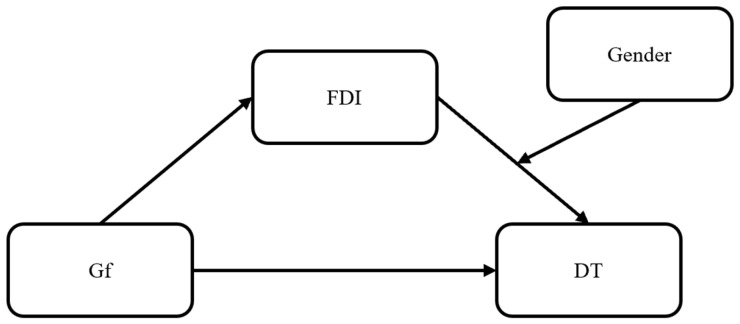
The moderated mediation model proposed in the current research. Note. Gf = Fluid intelligence; FDI = Field dependent-independent cognitive style; DT = Divergent thinking.

Based on these premises, the present study advanced a moderated mediation model where Gf was the independent variable, DT the dependent variable, FDI the mediator, and gender was the moderator (Figure 1). Hereafter the hypotheses follow:

**Hypothesis 1 (H1).**  *FDI mediates the association between Gf and DT.*

**Hypothesis 2 (H2).**  *Gender moderates the association between FDI and DT, enhancing this association when boys are involved.*

## 2. Materials and Methods

### 2.1. Participants

An a priori power analysis through G*Power 3.1 [53] has been computed to identify the minimum sample size. In line with Cohen [54], the following parameters have been entered in the software G*Power: test family: “*F* test analysis”, statistical test: “Linear multiple regression: fixed model, *R*^2^ deviation from zero”, type of analysis: “A priori: Compute required sample size—given *α*, power and effect size”, *α* err prob = 0.05, power (1-*β* err prob) = 0.95, mean effect size *f*^2^ = 0.15 (medium effect), and a maximum number of predictors = 5. G*Power suggested a recommended minimum sample size of N = 92. In total, 101 school-age children (mean_age_ = 8.02 years; SD_age_ = 1.43 years; range_age_ = 6–10 years; 50 girls) have been enrolled in current research. All children have been recruited from a local family pediatrician in L’Aquila, Abruzzo region (Italy). All children were Italian speakers and had no neurodevelopmental diseases, learning difficulties, and primary visual or hearing impairments, and emotional or behavioral problems (as reported by pediatrician anamnesis).

### 2.2. Measures

#### 2.2.1. Fluid Intelligence (Gf)

**Figure 2:**
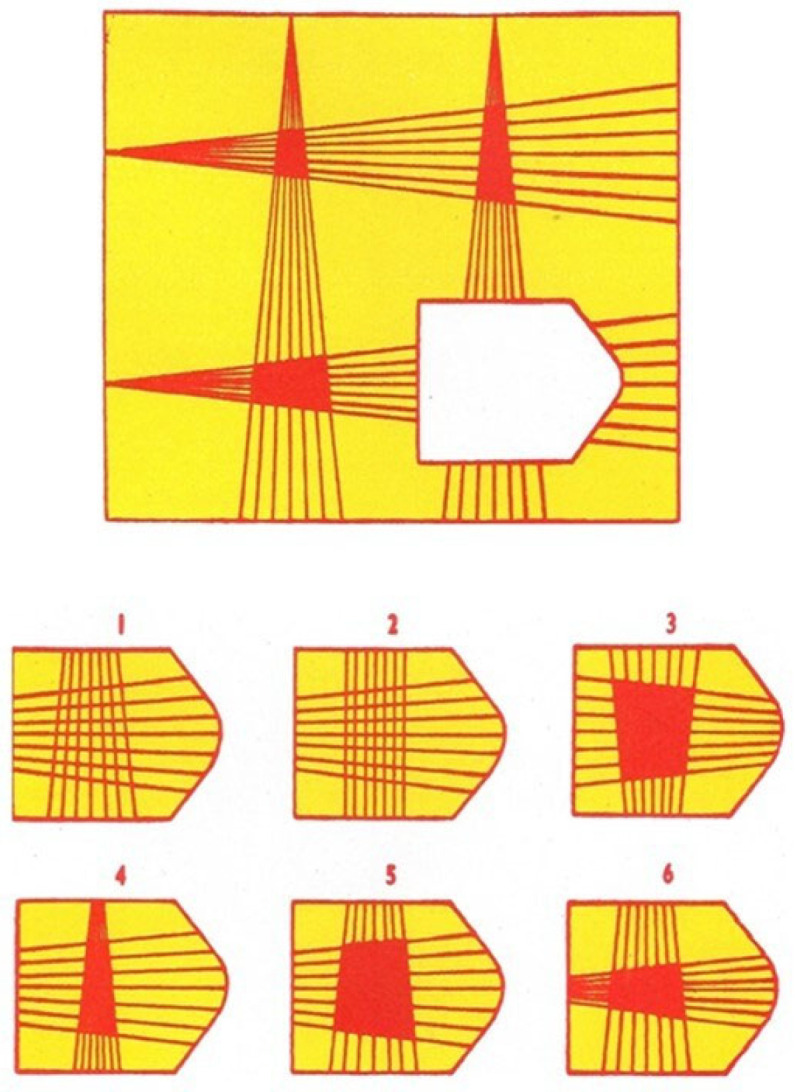
Item No.12 of the Coloured Progressive Matrices.

The Colored Progressive Matrices (CPM; [55]) evaluates Gf in children by three different series (12 items per series). Each item depicts a figure with specific relations among elements. The figure includes also a missing graphic element. Participants are required to indicate which one of the six graphic elements displayed below the main figure is suitable to complete the figure (see Figure 2). The maximum score is 36. As shown in the technical manual, the CPM demonstrated very good psychometric properties with a test–retest reliability typically around 0.90 and a Cronbach’s alpha of 0.91. Additionally, the CPM showed a strong convergent validity (*r* ranging from 0.60 to 0.90) [55].

#### 2.2.2. Field Dependent-Independent Cognitive Style (FDI)

**Figure 3:**
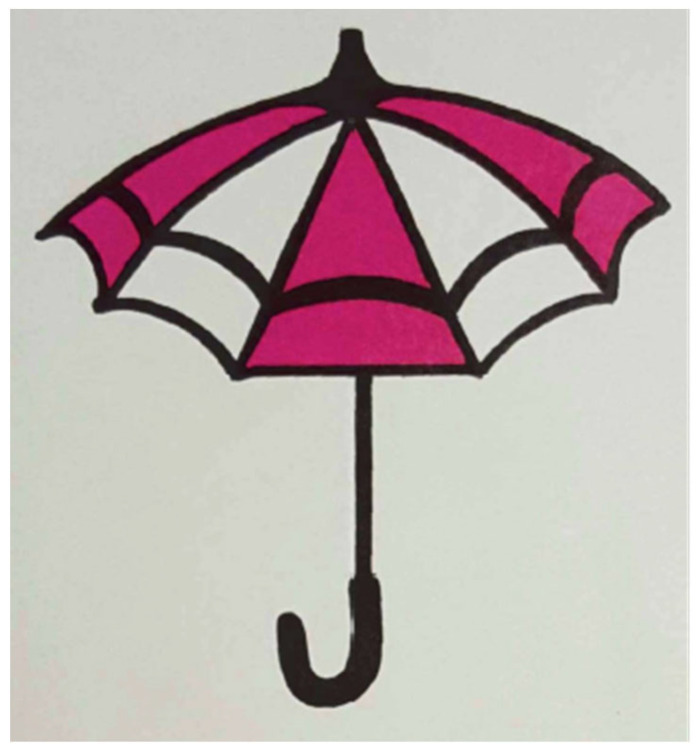
An example of large complex colored figure of the CEFT.

The Children’s Embedded Figure Test (CEFT; [56]) assesses children’s disposition toward FDI. The CEFT consists of 25 colored testing items (Figure 3). Participants are requested to find a simple figure within a more complex one. No time limit was given to complete the task. Accuracy was collected. The higher number of correct responses indicates the higher disposition toward field independence. The CEFT showed moderate internal consistency (0.86), and the test–retest reliability (after 1 year) was about 0.63 [43].

#### 2.2.3. Divergent Thinking (DT)

The Alternative Uses Task (AUT) from the Torrance Test of Creative Thinking TTCT-Form A [57] allows for assessing DT and requests to find as many uses as possible for carton boxes. Participants were requested to complete the task within 10 min. DT has been evaluated by two trained judges (2 females: M_age_ = 20 years; SD_age_ = 1.41 years) considering the following parameters: (1) fluency, that is, the number of appropriate and useful alternative uses (DT-Fluency); and (2) creativity. This latter criterion has been assessed using the snapshot scoring method widely used in previous research on DT [58]. The snapshot scoring method consists of computing a single score of creativity by judging the entire set of responses by weighting on a 5-point Likert-type scale (from 1 = low creativity to 5 = high creativity) in terms of uncommonness, remoteness, and cleverness. Specifically, uncommonness reflects the degree of infrequency of the ideas generated, remoteness denotes the extent to which the idea generated is remotely linked to everyday objects, and cleverness entails the degree of insightful, humor, irony, and smartness of ideas [58]. The evaluations provided by the two judges have been averaged to compute a DT creativity score (DT-Creativity; inter-rater correlations: *α* = 0.91, *p* < 0.001).

### 2.3. Procedure

A local pediatrician informed the children’s parents about the possibility of participating in the study. If parents agreed, an email was sent to them detailing the research procedures and the written consent. Individual testing was envisaged in a room of the pediatric clinic. This study was included in a research project on children’s cognitive functioning. Participation was voluntary. The study has been conducted in accordance with the Declaration of Helsinki. The research was ethically endorsed by the Research Ethics Committee of the University of L’Aquila (prot. n. 11/2020) on the 21 April 2020.

### 2.4. Statistics

Data has been analyzed using SPSS Statistics version 24 for Windows (IBM Corporation, Armonk, New York, USA). The main features, as well as the association among the study variable, have been preliminarily analyzed through descriptive statistics and correlations. The PROCESS macro for SPSS has been used for testing the mediation of FDI and the moderation of gender (version 3.5) [59]. Mediation and moderation have been computed using 95% confidence interval (CI) bootstrapped based on 5000 samples. The bootstrapping approach allows for a reliable evaluation of the mediating and/or moderating effects also in small as well as medium sized samples [60,61]. The significance of the mediating and/or moderating effect is provided if the range of the bootstrapped CI does not include the value of zero [62]. In a bootstrapping approach, the *R*^2^ is used as a measure of the effect size. All significance was set to *p* < 0.05.

## 3. Results

### 3.1. Preliminary Analysis

The Kolmogorov–Smirnov Test indicated that data were not normally distributed except for Gf (Kolmogorov–Smirnov Test: Z_Aage_ = 0.12, *sig*; Z_FDI_ = 0.00, *sig*; Z_DT-Creativity_ = 0.02, *sig*; Z_Gf_ = 0.13, *ns*). In addition, the z-test revealed no univariate outliers (cut-off ± 3). As shown in Table 1, correlational analysis indicated that age was positively correlated with Gf (*r* = 0.65; *p* < 0.01), FDI (*r* = 0.63; *p* < 0.01), and DT-Creativity (*r* = 0.23; *p* < 0.05). In addition, Gf was positively correlated with FDI (*r* = 0.65; *p* < 0.01) and DT-Creativity (*r* = 0.31; *p* < 0.01). Finally, this latter was positively correlated with FDI (*r* = 0.42; *p* < 0.01).

### 3.2. Testing for the Mediating Effect of Field Dependent-Independent Cognitive Style

Using the Model 4 of the PROCESS macro, results indicated that Gf was associated with FDI (b = 0.29, *p* < 0.001), which in turn was associated with DT-creativity (b = 0.14, *p* < 0.001). The direct effect of Gf on DT-creativity was not significant (b = 0.02, SE = 0.03, *p* > 0.05, 95% *CI* [−0.028, 0.076]), whereas the indirect effect was significant (b = 0.04, SE = 0.01, 95% *CI* [0.019, 0.065]). Overall, these findings confirmed H1, suggesting that FDI fully mediated the association between Gf and DT.

### 3.3. Testing for the Moderating Effect of Gender

Using the Model 14 of the PROCESS macro, results indicated that the association between FDI and DT was moderated by gender (b = 0.14, SE = 0.05, t = 2.84, 95% *CI* [0.040, 0.230]) (Table 2) considering both boys (b = 0.22, SE = 0.04, t = 5.01, 95% *CI* [0.134, 0.310]) and girls (b = 0.09, SE = 0.03, t = 2.76, 95% *CI* [0.024, 0.149]) (Table 3). Overall, these findings confirmed H2, indicating that the effect of FDI on DT was stronger among boys.

### 3.4. Post Hoc Power Analysis

In order to evaluate the power obtained from the collected data, we performed a post hoc power analysis, which indicated that the power value for the moderated mediation model was 1.00. Therefore, the research sample was appropriate and satisfies the recommended cut-off value of 0.80 [63].

## 4. Discussion

The present study addressed the mediating role of FDI in the interplay between Gf and DT in school-age children, exploring also the moderating effect of gender in the association between FDI and DT. This study moves from the evidence that, in both adolescents and young adults, analytical reasoning implicated in the field independent cognitive style is critical in the association between Gf on DT [35,36]. In order to understand if such a chain of relationships originates since childhood, the idea was to replicate previous results in elementary school-age children. Additionally, the potential involvement of gender as a moderator was also considered, as previous studies showed that boys are more field independent than girls [49,50]. Thus, given that the interplay between Gf, FDI, DT and gender have never been studied in a sample of children using a moderated mediation approach, this study is unique in that.

The results confirmed H1, revealing that the association between Gf and DT was fully mediated by FDI, suggesting that FDI is critical in transmitting the effect of Gf on the ability to think divergently. Specifically, the higher Gf the higher the children’s disposition toward the field independence cognitive style. In addition, the higher children’s field independence the higher the ability to think divergently. These findings suggested that field independence reflects Gf controlled processes, which allow for setting up goals and execute actions [36]. This disposition supports ill-defined problem solving (i.e., finding alternative uses of an object) through a more analytical strategy, which are helpful to breaking down the problem into steps and reaching creative solutions successfully [35]. The impact of the field independence cognitive style in the association between Gf and DT seems to change when age increases. Indeed, whereas in childhood and adolescence, FDI fully mediated the relationship between Gf and DT, in adulthood, it is implicated only partially [35,36]. This evidence suggests that the enhancement of Gf from childhood to early adulthood [64] could change the weight of the contribution of one’s resources in solving ill-defined problems, such as those underpinning DT. During adulthood, thinking divergently could require not only information processing strategies to process and organize information from the surrounding environment (i.e., FDI), but also a higher evaluation of ideas according to effectiveness, future usability, and functionality. These abilities are supported by Gf [16,17]. Therefore, it is possible that reasoning and problem-solving abilities reflecting Gf were not found to directly predict DT because they are not adequately developed in early age of life. Instead, by cerebral maturation and accumulated experience, these abilities are well developed in adulthood and consequently can directly predict DT [36].

Additionally, our results revealed that gender moderated the interplay between FDI and DT. Notably, even though the moderating effect was found for either boys or girls, the FDI-DT link was stronger among boys, supporting H2. This result confirms that autonomy and analytic thinking involved in field independence are strengthened in boys, even though it is not possible to rule out the possibilities that also girls develop such abilities to some extent. Probably, this result could be explained considering the requests of the current socio-cultural and educational context. Indeed, today’s child, regardless of gender, is usually involved in activities requiring analytical thinking, autonomy and related skills since the early stages of life [65]. For example, in both informal (e.g., family) and school contexts, children are exposed since pre-school age not only to socio-emotional stimulations, but also to science, technology, engineering and mathematics (STEM) learning, which usually requests to break down different types of problems that include various epistemologies, claims, and content into small parts and find potential relationships: the development of these abilities is useful to meet school expectations [66].

The present study shows several implications. Given that FDI is critical in the association between children’s Gf and DT, stimulation programs aimed at promoting DT based on the field independence cognitive style could be developed since the early ages. In this vein, knowing that boy’s higher field-independence increases DT is useful to tailor stimulation programs. Of course, the opportunity to better select children according to their abilities and predispositions might be pursuit in school contexts to promote a set of abilities, such as creativity and analytical thinking, that are crucial for a positive development in terms of school and academic success as well as future jobs and, ultimately, everyday life [67].

This study is not without limitations. First, given the cross-sectional nature of the present research, which does not allow for causal inferences, longitudinal designs should be envisaged in future studies. This allows for better exploring the mediating role of FDI and the moderating effect of gender. Second, in the current research, only FDI has been detected. To better explore the mediating role of cognitive styles, future studies should consider a more comprehensive evaluation of the realm of cognitive styles [68], such as impulsiveness vs. reflection, global vs. local, and flexible vs. normative should be envisaged. Third, the current study focused only on a single facet of EFs, that is, Gf. To provide a more comprehensive evaluation of the impact of executive functioning on DT, future research should also consider other EFs, such as planning, working memory, inhibition, and flexibility [69]. Fourth, socio-economic status information was not collected. Since this variable is involved in developmental processes even of creativity [70], it is important to control for such a factor in further research.

## Figures and Tables

**Table 1:** Descriptive statistics and correlations among all study variables.

	M	SD	1.	2.	3.	4.	5.
1. Age	8.02	1.43	1				
2. Gender			0.00	1			
3. Gf	25.88	4.71	0.65 **	0.06	1		
4. FDI	20.70	3.83	0.63 **	0.05	0.65 **	1	
5. DT-Creativity	2.44	1.03	0.23 *	0.33 **	0.31 **	0.42 **	1

Note. N = 101, Gender was dummy coded (0 = F; 1 = M). Gf = Fluid intelligence; FDI = field dependent-independent cognitive style; DT = divergent thinking. ** *p* < 0.01 (two tailed). * *p* < 0.05 (two tailed).

**Table 2:** Main and interaction effects of the moderated mediation model.

Predictors	b	SE	t	Boot LLCI	Boot ULCI
Gf	0.02	0.02	0.73	−0.030	0.065
FDI	0.09	0.03	2.76	0.024	0.149
Gender	0.58	0.16	3.53	0.253	0.904
FDI × Gender	0.14	0.05	2.84	0.040	0.230
Age	−0.06	0.08	−0.71	−0.217	0.103
*R* ^2^	0.41				
*F*(5,95)	13.06 ***				

Note. N = 101. Gf = Fluid intelligence; FDI = Field dependent-independent cognitive style; DT = Divergent thinking. *** *p* < 0.001.

**Table 3:** Conditional indirect effect of gender on DT.

	b	Boot SE	Boot LLCI	Boot ULCI
Girls	0.02	0.01	0.006	0.044
Boys	0.06	0.02	0.030	0.104
Index of moderate mediation	0.04	0.02	0.008	0.104

## Data Availability

The data presented in this study are available on request from the corresponding author. The data are not publicly available due to specific ethical and privacy considerations.
